# *In situ* growth of CuS nanoparticles on g-C_3_N_4_ nanosheets for H_2_ production and the degradation of organic pollutant under visible-light irradiation†

**DOI:** 10.1039/c9ra03532j

**Published:** 2019-08-15

**Authors:** Zhenhe Xu, Baotong Xu, Kun Qian, Zheng Li, Fu Ding, Miaomiao Fan, Yaguang Sun, Yu Gao

**Affiliations:** The Key Laboratory of Inorganic Molecule-Based Chemistry of Liaoning Province, College of Applied Chemistry, Shenyang University of Chemical Technology Shenyang 110142 China xuzh@syuct.edu.cn dingfu@syuct.edu.cn gaoy777@126.com; Liaoyang Institute for Drug Control Liaoyang 111000 China

## Abstract

The solar-to-fuel conversion using a photocatalyst is an ideal method to solve the energy crisis and global warming. In this contribution, photocatalytic H_2_ production and organic pollutant removal using g-C_3_N_4_/CuS composite was demonstrated. Well dispersed CuS nanoparticles (NPs) with a size of about 10 nm were successfully grown on the surface of g-C_3_N_4_ nanosheet *via* a facile hydrothermal method. The as-prepared g-C_3_N_4_/CuS nanocomposite at an optimized loading exhibited a much higher visible light photoactivity, giving up to 2.7 times and 1.5 times enhancements in comparison to pure g-C_3_N_4_ for photocatalytic H_2_ production and methylene orange (MO) degradation, respectively. These enhanced photocatalytic activities are attributed to the interfacial transfer of photogenerated electrons and holes between g-C_3_N_4_ and CuS, which leads to effective charge separation on both parts. That is, under the visible light irradiation, electrons in the valence band (VB) of g-C_3_N_4_ can directly transfer to the CuS NPs, which can act as an electron sink and co-catalyst to promote the separation and transfer of photo-generated electrons, thus significantly improving the photocatalytic efficiency.

## Abstract 2

A novel ternary g-C_3_N_4_/CuS photocatalyst shows a good photocatalytic H_2_ production and methylene orange degradation under visible light.

## Graphical abstract

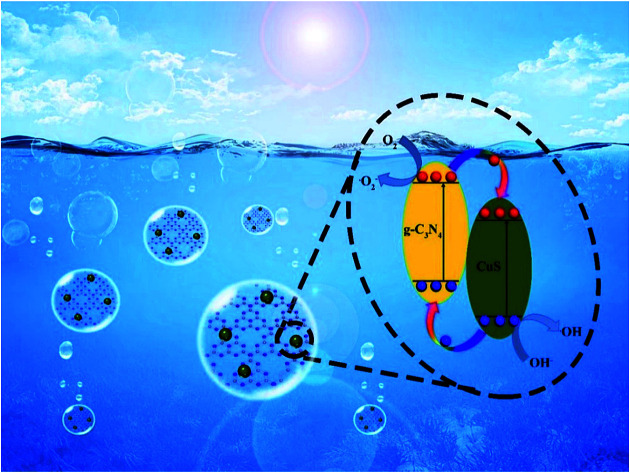

## 1. Introduction

Developing and using renewable energy sources is believed to be an effective approach to solve the environmental and energy challenges facing the world in the 21st century arising from the overuse of fossil fuels and the resulting serious environmental pollution problems.^1–3^ Solar-light driven photocatalytic photocatalysis has been regarded as an ideal “green strategy” for environmental remediation and energy conversion.^4,5^ Furthermore, the potential success of this strategy relies largely on the development of semiconductor materials which have some key requirements, including: the material should be able to absorb visible light to maximize use of the solar spectrum, and the electrons and holes will migrate to reactive sites instead of recombining with each other. The material should also be abundant, cheap, non-toxic, and stable in different reaction environments.^6–8^

Polymeric graphitic carbon nitride (g-C_3_N_4_) is a paradigm photocatalyst due to its unique electric, optical, structural, and physiochemical properties.^9–11^ State-of-the art progress has been achieved in the photocatalysis over g-C_3_N_4_ semiconductor materials since Wang and his co-workers first reported the photocatalytic H_2_ evolution over g-C_3_N_4_ in 2009.^12^ Unfortunately, a single g-C_3_N_4_ alone is very difficult to satisfy the practical application because of the ultrafast electron–hole recombination rate, low surface area of g-C_3_N_4_ (∼10 m^2^ g^−1^), and small surface active sites for sluggish uphill H_2_-evolution reactions.^13^ In these circumstances, ever more endeavors are devoted to developing g-C_3_N_4_-based semiconductor materials through different thermodynamic (*e.g.*, doping and sensitization) and kinetics (*e.g.*, constructing heterojunctions, *Z*-scheme systems, fabricating micro/nano architectures, and loading proper cocatalysts) methods.^10,11,14^ In recent years, researchers have carried out a lot of research in this area. For example, Cui *et al.* developed a facile and in-air chemical vapor deposition (CVD) method that produces onion-ring-like g-C_3_N_4_ microstructures.^15^ The optimal synthesized sample (RCN-350) shows a high H_2_ evolution rate of 1900.0 μmol h^−1^ g^−1^ under visible light irradiation. The g-C_3_N_4_/V_o_-ZnO hybrid photocatalyst with a g-C_3_N_4_ content of 1 wt% exhibited enhanced photocatalytic activity for degradation of organic contaminants than pure V_o_-ZnO and g-C_3_N_4_ under visible light irradiation.^16^ Very recently, Xu *et al.* firstly reported that the photocatalytic activity of 1,1′-bis(4-carboxylatobenzyl)-4,4′-bipyridinium dichloride (denoted as CBV^2+^) coupled with g-C_3_N_4_ through hydrogen bonds.^17^ When 1 wt% CBV^2+^ is introduced, the hydrogen production rate of g-C_3_N_4_/CBV^2+^ dramatically increases up to 41.57 μmol h^−1^, exceeding 85 times the rate over bare g-C_3_N_4_ (only 0.49 μmol h^−1^).

Among above mentioned various kinds of methods, loading proper cocatalysts is extensively considered one effective approach to improve the photocatalytic behavior of g-C_3_N_4_-based photocatalysts, which can simultaneously achieve the promoted charge separation, accelerated surface reaction kinetics, and suppressed surface back reactions. Up to now, many heterostructures such as g-C_3_N_4_/Ag, g-C_3_N_4_/AgCl,^18^ g-C_3_N_4_/Bi_2_WO_6_, g-C_3_N_4_/black phosphorus,^19^ have been reported to be active photocatalysts and were found to show excellent photocatalytic activity toward the decomposition of organic pollutants and H_2_ generation from water splitting under visible-light illumination. Compared to the low natural abundance and high cost of noble metals, the earth-abundant metals and their compounds seem to be more promising for practical applications on a large scale. Copper sulfide (CuS), with a relatively narrow band gap, is a nontoxicity, good photosensitivity, and excellent physical and chemical stability, eco-friendly and economic suitable materials, which has gained immense interest.^20^ Furthermore, the energy levels of CuS and g-C_3_N_4_ match and overlap each other to construct the heterojunction, thereby showing excellent visible-light photocatalytic activity.

In the present study, we report a visible light-driven photocatalytic H_2_ production and organic pollutant removal based on g-C_3_N_4_/CuS material prepared by a simple *in situ* growth hydrothermal method. The as-prepared nanocomposite at an optimized loading exhibited a much higher visible light photoactivity, giving up to 2.7 times and 1.5 times enhancements in comparison to pure g-C_3_N_4_ for photocatalytic H_2_ production and methylene orange (MO) degradation, respectively. Furthermore, the possible underlying mechanisms were proposed. This study may open a new avenue for developing high efficiency, non-toxic, and low-cost g-C_3_N_4_-based photocatalysts for the visible-light photocatalysis.

## 2. Experimental section

### 2.1 Materials

Urea, copper(ii) nitrate trihydrate (Cu(NO_3_)_2_·3H_2_O), thioacetamide (TAA), methyl orange (MO), 1,4-benzoquinone (BQ), disodium triethylamine (TEA), and *tert*-butyl alcohol (*t*-BuOH) were purchased from Aladdin Reagent Co. Ltd. All chemicals were analytically pure and used without further purification. The absolute pure water, purified by a Millipore Ultrapure water system and having a resistivity of 18.2 MΩ cm at 25 °C, was used in the current investigation.

### 2.2 Synthesis of the g-C_3_N_4_/CuS nanocomposites

**Scheme 1:**
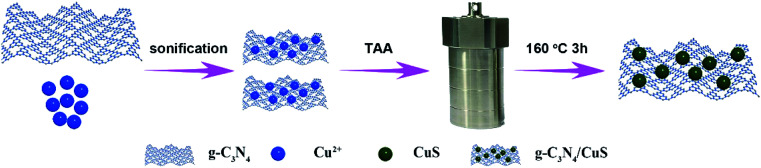
Schematic illustration for the synthesis of the g-C_3_N_4_/CuS photocatalyst.

The synthesis procedures of g-C_3_N_4_/CuS nanocomposites are shown in Scheme 1. 30 g of urea was placed into an alumina crucible with a cover, and then the crucible was heated in a muffle furnace at 550 °C for 3 h with a heating rate of 1 °C min^−1^. The yielded yellow powder was washed with nitric acid (0.1 mol l^−1^) and distilled water to remove any residual alkaline species (*e.g.* ammonia) adsorbed on the surface of the product, and then the product was dried at 80 °C for 12 h. The g-C_3_N_4_/CuS nanocomposites were prepared by a hydrothermal method. Typically, 0.25 g of g-C_3_N_4_ was dispersed into 20 ml of deionized water under ultrasonication for 0.5 h, and then a certain amount of Cu(NO_3_)_2_·3H_2_O and TAA were added slowly to the dispersion with the same molar of Cu^2+^ and S^2−^. After ultrasonication for another 0.5 h, the mixture was transferred into a 100 ml a Teflon bottle held in a stainless-steel autoclave, sealed, and maintained at 160 °C for 3 h. After naturally cooling down to room temperature, the solid product was collected and washed thoroughly by centrifugation with deionized water, and then dried at 60 °C for 12 h. According to this method, different weight ratios of g-C_3_N_4_/CuS samples with CuS contents of 0.25 wt%, 0.5 wt%, 0.75 wt%, and 1.0 wt% were synthesized. Pure CuS was prepared similarly without introducing g-C_3_N_4_.

### 2.3 Characterization

The X-ray diffraction (XRD) patterns of the samples were recorded on a D8 Focus diffractometer (Bruker) with use of Cu Kα radiation (*λ* = 0.154 nm). The microstructure and composition of the photocatalysts were studied by a transmission electron microscope (TEM, JEOL 2100F, operated at 200 kV), combined with energy-dispersive X-ray (EDX) spectroscopy. The UV-vis diffuse reflectance spectra (DRS) were recorded on a UV-vis spectrometer (UV-2550, Shimadzu, Japan) equipped with an integrating sphere. The photoluminescence (PL) spectra were measured by using a fluorescence spectrophotometer (F4600, Hitachi, Japan) with the excitation wavelength of 300 nm.

### 2.4 Photocatalytic hydrogen evolution

The photocatalytic hydrogen production reactions were performed in Perfect Light Labsolar-6A automatic online photocatalytic analysis system (Labsolar-6A, Beijing Perfect light Co., Ltd.). In a typical experiment, 50 mg photocatalyst was well dispersed in a 100 ml aqueous solution containing 10 ml of triethanolamine as the sacrificial reagent. The reactant solution and the system were thoroughly evacuated several times to insure the complete removal of the air. The photocatalytic reaction was triggered by the visible-light irradiation offered by a 300 W Xe lamp (PLS-SXE 300 W xenon lamp Beijing Perfect light Co., Ltd.) with a 420 nm cut-off filter (Fig. S1†) under continuous stirring. The temperature of the reaction solution was maintained at room temperature by a water-cooling system. The amount of evolved H_2_ was analyzed by an online gas chromatograph (Shimadzu GC-2014) equipped with a thermal conductivity detector and high-purity Ar carrier gas was used to analyze reaction-evolved gases.

### 2.5 Photodegradation of MO and the detection of active species

The photodegradation performances of the as-prepared g-C_3_N_4_/CuS photocatalysts were investigated by photodegrading MO dye in aqueous solution. 10 mg of the as-prepared photocatalysts were added into 20 ml of MO (10 mg l^−1^) solution in a 50 ml quartz reactor with circulating cooling water to keep the reaction temperature constant. Before illumination, the mixed suspension was magnetically stirred in the dark for 30 min to obtain the adsorption–desorption equilibrium. A 300 W xenon lamp filtered by a UV cut-off filter (*λ* > 420 nm) was used as the visible light source. At certain time intervals, 0.5 ml of the reaction solution was taken out and centrifuged to remove the catalyst, then analyzed on UV-vis spectrometer to detect the residual concentration of MO in the solution. In addition, in order to detect the generated active species in the photocatalysis, 1,4-benzoquinone (BQ) (1 mM), disodium ethylenediaminetetraacetate (Na_2_EDTA) (1 mM), and *tert*-butyl alcohol (*t*-BuOH) (1 mM) were used as superoxide radical (·O_2_^−^), hole, and hydroxyl radical (·OH) scavengers, respectively, with all other conditions being the same.

### 2.6 Electrochemical analysis

The photoelectrochemical (PEC) measurements were performed with an electrochemical workstation (CHI 660E, CH Instruments) in a standard three electrode cell, using a Pt wire and a Ag/AgCl electrode (3 M KCl) as the counter and reference electrode, respectively. The working electrode was prepared on fluorine-doped tin oxide (FTO) glass with its boundary being protected by Scotch tape. Five milligrams of as-synthesized powder was dispersed into 1 ml of dimethylformamide under sonication for 30 min to get a colloidal dispersion. The dispersion was drop-casted onto the FTO glass. After natural air drying, the uncoated part of the FTO glass was isolated with epoxy resin glue. The 0.2 M of Na_2_SO_4_ (pH = 6.8) aqueous solution prepurged with nitrogen for 30 min was used as an electrolyte. A solar simulator was utilized as a light source for the measurements. Nyquist plots were recorded over the frequency range of 100 mHz to 100 kHz at a bias of 0.2 V.

## 3. Results and discussion

### 3.1 Characterization

**Fig. 1:**
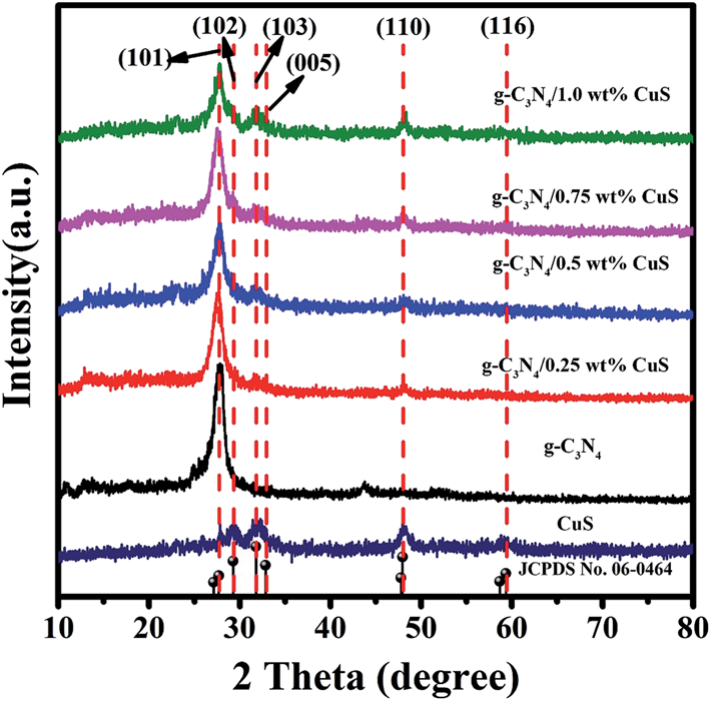
XRD patterns of CuS, g-C_3_N_4_, g-C_3_N_4_/*x* wt% CuS (*x* = 0.25, 0.5, 0.75, and 1.0).

The crystallinity and phase purity of the as-prepared samples were characterized by XRD (Fig. 1). For the pure CuS, it can be seen that all the peaks of the sample can be readily indexed to a pure hexagonal phase CuS according to the JCPDS card no. 06-0464 (*a* = *b* = 3.792 Å and *c* = 16.344 Å). As for the pure g-C_3_N_4_ sample, it shows diffraction peaks at 13.1° and 27.4°, which can be indexed as the (100) crystal plane of tri-*s*-triazine units and the (002) diffraction for interlayer stacking of aromatic systems of graphitic materials, respectively. For the g-C_3_N_4_/CuS samples, all the samples show quite similar profiles, that is, the XRD patterns show g-C_3_N_4_ and CuS phases. With an increasing amount of CuS from 0.25 wt% to 1.0 wt%, the diffraction peaks of CuS are intensified gradually, whereas the intensities of the peaks of g-C_3_N_4_ are weakened. These results indicated that CuS NPs were successfully loaded on the g-C_3_N_4_ by the present synthetic route.

**Fig. 2:**
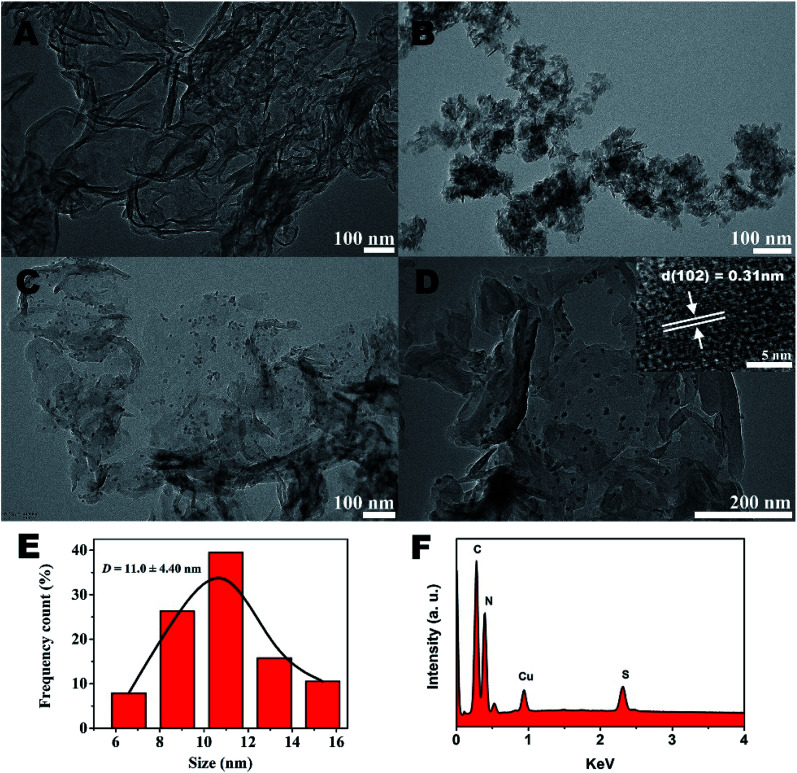
TEM images of (A) pure g-C_3_N_4_, (B) pure CuS, (C) g-C_3_N_4_/CuS. (D) The EDX of g-C_3_N_4_/CuS sample. The inset in (C) is the HR-TEM image of the g-C_3_N_4_/CuS sample. (E) The size distribution profile of the CuS NPs. (F) The EDX of g-C_3_N_4_/CuS sample.

The further morphology and microstructure of the as-prepared samples were analyzed by transmission electron microscopy (TEM) technique in Fig. 2. The typical TEM image (Fig. 2A) of g-C_3_N_4_ shows 2D sheet-like nanostructures with transparent thin layers resembling the graphene nanosheets. For CuS sample (Fig. 2B), irregularly aggregation can be observed. However, surprisingly, by the hydrothermal method to prepare g-C_3_N_4_/CuS nanocomposite, the TEM result (Fig. 2C) clearly confirmed the successful loading of CuS NPs onto the g-C_3_N_4_ nanosheet surface, and no free CuS NPs were present in the suspension. A higher magnification TEM image (Fig. 2D) clearly demonstrate that the average size of CuS NPs is around 11.0 ± 4.4 nm (Fig. 2E). The HRTEM image (inset in Fig. 2D) shows that the lattice fringes with *d*-spacing of 0.31 nm can be assigned to the (102) crystal plane of hexagonal CuS phase. These results indicate that the g-C_3_N_4_ nanosheets significantly influence the growth of CuS NPs and effectively restrain their aggregations. It could be attributed to two possible reasons. First, the g-C_3_N_4_ nanosheet may act as a two-dimensional “mat” that interacts with CuS NPs through physisorption to hinder their aggregation. Second, the oxygen-containing defects and the amino groups on g-C_3_N_4_ surface could serve as anchor sites to immobilize Cu^2+^ ions as well as CuS NPs on g-C_3_N_4_ nanosheet, which prevents the aggregation of CuS NPs from surface diffusion on g-C_3_N_4_ nanosheet.^21^ The energy dispersive X-ray (EDX) results explicitly illustrated the existence of C, N, Cu and S elements in the region (Fig. 2F).

**Fig. 3:**
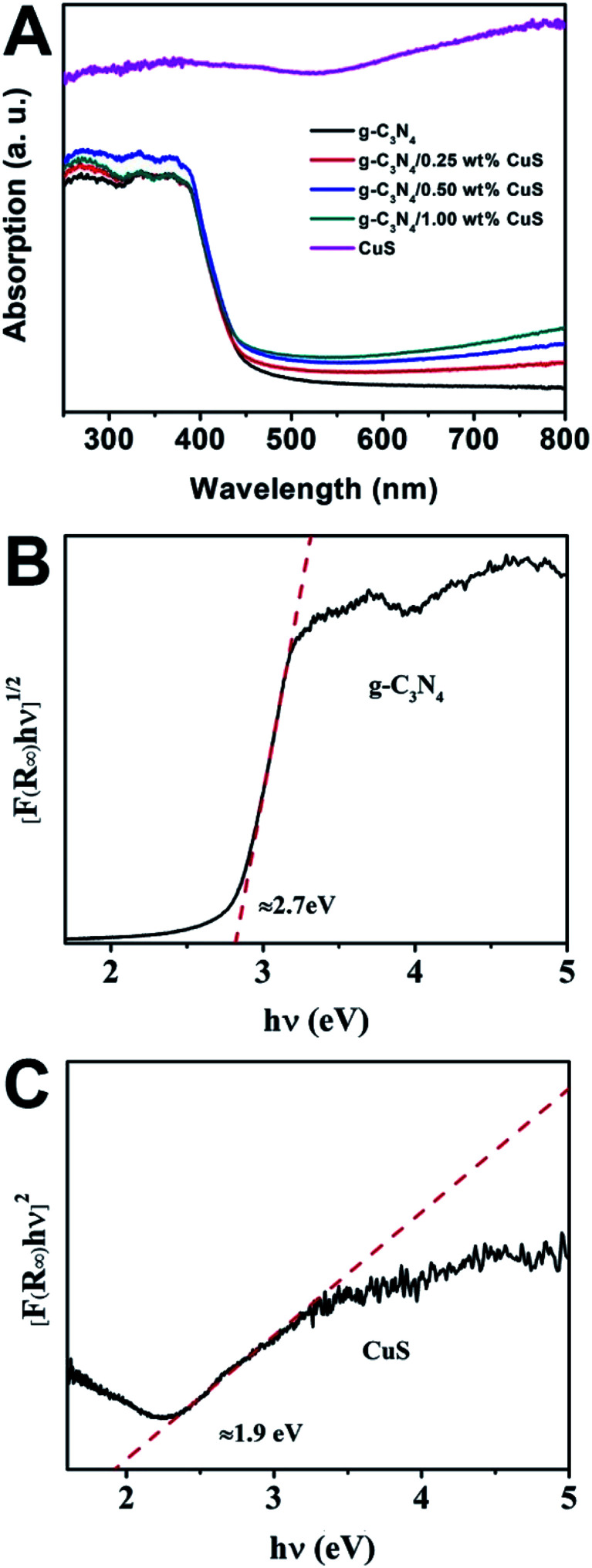
(A) UV-vis diffuse reflectance spectra of CuS, g-C_3_N_4_, g-C_3_N_4_/*x* wt% CuS (*x* = 0.25, 0.5, and 1.0). The plot of (*αhv*)^2^*versus* energy (*hv*) for the band gap energy of (B) g-C_3_N_4_ and (C) CuS.

The UV-vis spectra were used to study the optical character of the as-obtained samples. As shown in Fig. 3A, the pure g-C_3_N_4_ displays intense absorption bands with absorption edges at around 450 nm, suggesting a band gap of about 2.70 eV. When the CuS NPs were loaded on the g-C_3_N_4_ nanosheet, intensities of the absorption in the visible light region (*λ* > 450 nm) are gradually enhanced with increasing the CuS content. This enhancement is ascribed that pure CuS has an obvious wide absorption range from 300 nm to 800 nm. Thus, the presence of CuS NPs in the composites can reduce reflection of light and enhance the absorption. What is more, the g-C_3_N_4_/CuS composites show almost the same absorption edge in comparison to the pure g-C_3_N_4_, indicating CuS is not incorporated into the lattice of g-C_3_N_4_ and only are deposited on it's surface. We also estimated the band gap energies (*E*_g_) of g-C_3_N_4_ CuS by using the Kubelka–Munk transformation:^22^*αhv* = *A*(*hv* − *E*_g_)^*n*/2^where *α*, *h*, *v*, and *A* are the absorption coefficient, Planck constant, light frequency, and a constant. The *n* depends on the characteristics of the transition in a semiconductor. According to previous report, the values of g-C_3_N_4_ and CuS are 4 and 1, respectively.^23,24^ Thus, as displayed in Fig. 3B and C, the value of band gap for g-C_3_N_4_ and CuS are determined to be 2.7 eV and 1.9 eV, respectively. In addition, the potentials of valence band (*E*_VB_) and conduction band (*E*_CB_) of a semiconductor material could be calculated *via* the following empirical equations:*E*_VB_ = *X*_semiconductor_ − *E*^e^ + 0.5*E*_g_*E*_CB_ = *E*_VB_ − *E*_g_where *X*_semiconductor_ is the electronegativity of the semiconductor, *E*^e^ is the energy of free electrons *vs.* hydrogen (about 4.5 eV/NHE). The *X*_semiconductor_ values of g-C_3_N_4_ and CuS are 4.64 eV and 5.27 eV, respectively. So the *E*_VB_ and *E*_CB_ of g-C_3_N_4_ and CuS are 1.49 eV/NHE, −1.21 eV/NHE and 1.72 eV/NHE, −0.18 eV/NHE, respectively.

### 3.2 Photocatalytic performance

**Fig. 4:**
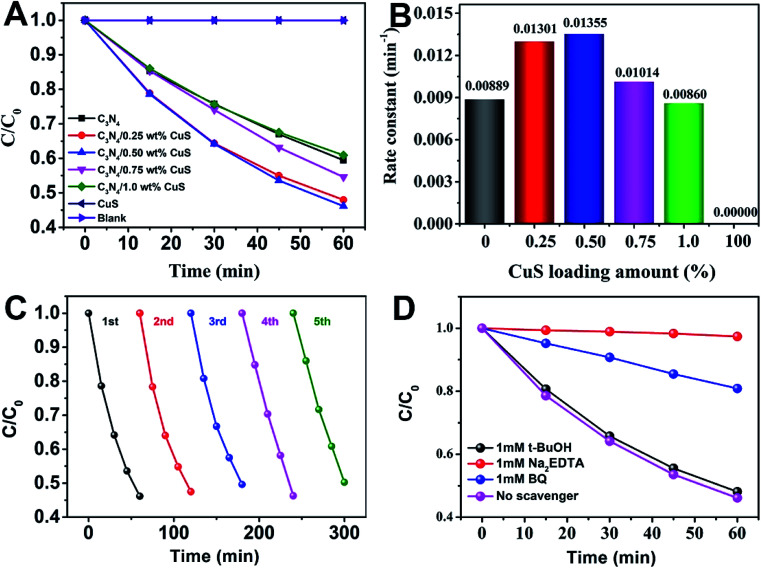
(A) The photodegradation activities of MO as a function of time over different photocatalysts under visible light irradiation (>420 nm), (B) the value of the rate constant *k* of the photodegradation of MO in the presence of as-prepared photocatalysts, (C) cycling runs of g-C_3_N_4_/0.5 wt% CuS photocatalyst in the photodegradation of MO under visible light irradiation (>420 nm), and (D) photodegradation of MO in the presence of three types of scavengers and g-C_3_N_4_/0.5 wt% CuS photocatalyst under visible light irradiation (>420 nm).

The photocatalytic activity of g-C_3_N_4_/CuS composites was first evaluated by hydrogen production reaction under visible light (*λ* > 420 nm), and the influence of photocatalysts containing different mass ratios of CuS on the H_2_ evolution is presented in Fig. 4. Fig. 4A shows the typical H_2_ evolution kinetics of different mass ratios of CuS under visible light irradiation. It should be noted that linearly increasing amounts of H_2_ evolved in all samples could be easily observed over the entire time range of light irradiation, confirming the relatively excellent photostabilities for all samples. There is a phenomenon is observed that no H_2_ can be detected when CuS alone is used as the photocatalyst, suggesting that pure CuS is not active for photocatalytic H_2_ production. However, CuS has a significant influence on the photocatalytic H_2_ production activity in the g-C_3_N_4_/CuS composites. The photocatalytic activities are gradually enhanced by increasing the CuS content, and it reaches a maximum value of 11.80 μmol h^−1^ over g-C_3_N_4_/0.50 wt% CuS sample, which is about 2.7 times higher than pure g-C_3_N_4_ (4.37 μmol h^−1^) (Fig. 4B). When CuS content is increased beyond 0.50 wt%, a decrease appeared in the photocatalytic H_2_ evolution results. Especially for g-C_3_N_4_/1.0 wt% CuS sample, the activity of H_2_ production decreases obviously, even slower than g-C_3_N_4_. The experiment on cyclic performance for H_2_ production was run under visible-light illumination (*λ* > 420 nm) for 5 consecutive cycles; no obvious decay of H_2_ production was observed, suggesting their quite good stability during water splitting (Fig. 4C).

**Fig. 5:**
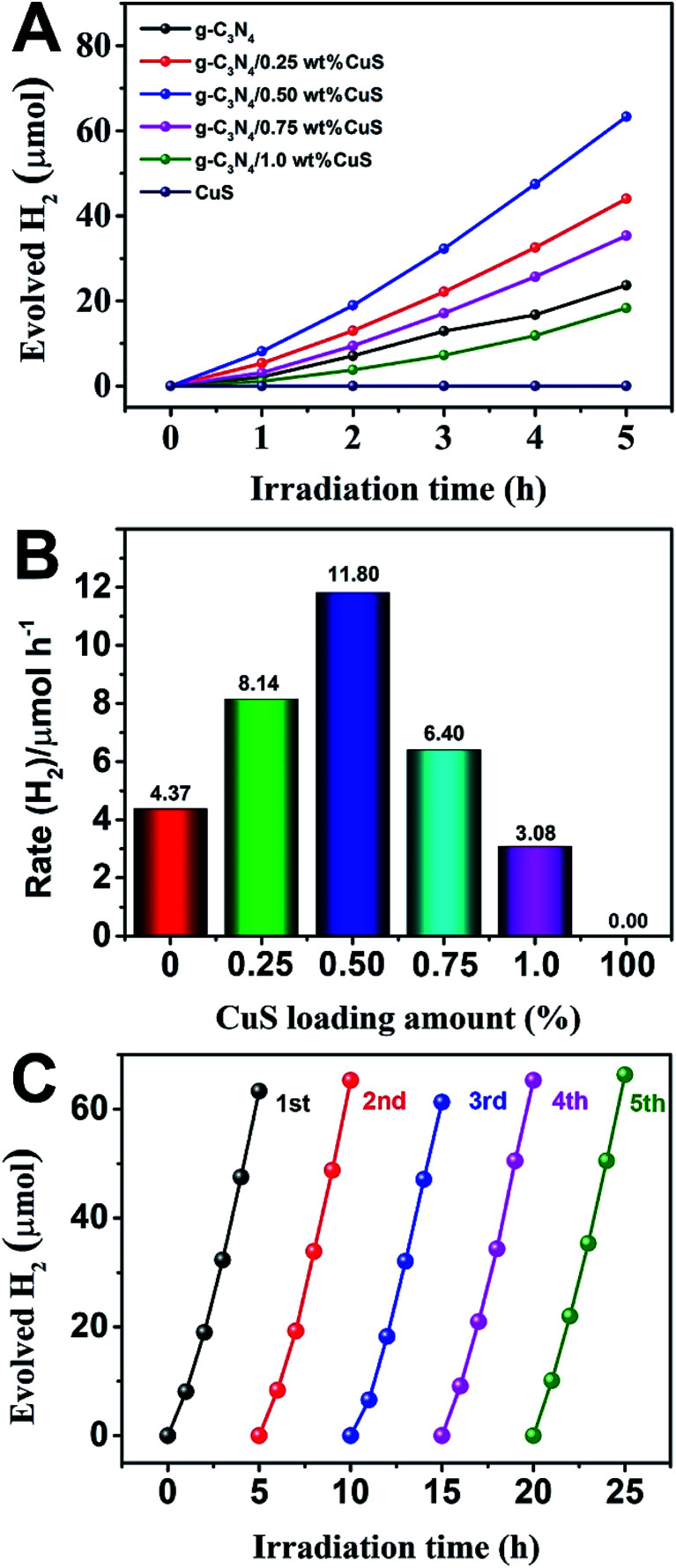
(A) Photocatalytic H_2_ production of the as-prepared photocatalysts under visible light irradiation (*λ* ≥ 420 nm). (B) The rate of H_2_ generation over the as-prepared photocatalysts. (C) Stability study of g-C_3_N_4_/0.5 wt% CuS for H_2_ evolution under visible light irradiation (*λ* ≥ 420 nm).

The photocatalytic activity of the g-C_3_N_4_/CuS composites was further assessed by the photodegradation of MO at room temperature under visible light (*λ* > 420 nm) irradiation (Fig. 5). MO was used as a target pollutant to evaluate the photocatalytic activity of the as-prepared samples because the photodegradation of MO is negligible under visible light, confirming that the photocatalytic activity indeed originates from the photocatalyst. Prior to irradiation, the suspensions were magnetically stirred in dark for 30 min to obtain the absorption–desorption equilibrium between the photocatalysts and MO. The variation in the absorption intensity of MO solution over CuS, C_3_N_4_, and g-C_3_N_4_/*x* wt% CuS (*x* = 0.25 wt%, 0.50 wt%, 0.75 wt%, and 1.0 wt%) at different irradiation times is recorded in Fig. S2 in ESI.† As shown in Fig. 5A, 0% and 40.5% MO are photodegraded by CuS and pure g-C_3_N_4_, respectively. While the g-C_3_N_4_/CuS composites show obviously enhance photocatalytic activities than pure g-C_3_N_4_ or CuS, especially the g-C_3_N_4_/0.50 wt% CuS shows the highest photodegradation ratio after 60 min, about 53.9% MO was decomposed. Furthermore, as the CuS content in the composites increases, the photocatalytic activity of the composite starts to decrease. The kinetic rate constants of the samples were obtained by plotting ln(*C*_0_/*C*) with the visible light irradiation time (minute), which were fitted with the pseudo-first-order model (Fig. 5B). The apparent rate constants calculated were 0.00889 min^−1^, 0.01301 min^−1^, 0.01355 min^−1^, 0.01014 min^−1^, 0.00860 min^−1^ and 0 min^−1^ for g-C_3_N_4_, g-C_3_N_4_/0.25 wt% CuS, g-C_3_N_4_/0.50 wt% CuS, g-C_3_N_4_/0.75 wt% CuS, g-C_3_N_4_/1.0 wt% CuS and CuS, respectively. The highest rate constant (0.01355 min^−1^) is achieved by the g-C_3_N_4_/0.50 wt% CuS, which is abut 1.5 times higher than the g-C_3_N_4_ (0.00889 min^−1^). Meanwhile, CuS doesn't show any visible light activity in the photocatalytic degradation of MO. To be served as a good photocatalyst, except for the enhanced visible light activity, the reusability and stability are also extremely important. The photocatalytic degradation of MO under visible light was carried out for 5 cycles in the presence of g-C_3_N_4_/0.50 wt% CuS photocatalyst (Fig. 5C). After 5 consecutive cycles, the g-C_3_N_4_/0.50 wt% CuS photocatalyst still shows very high photocatalytic degradation rate of MO. This result indicates that the as-prepared g-C_3_N_4_/CuS photocatalyst possesses excellent reusability and photostability.

In order to detect the main active species in the photocatalytic process, such as photogenerated holes, superoxide radicals (·O_2_^−^) and hydroxyl radicals (·OH), the trapping experiments in the presence of various scavengers were operated. As shown in Fig. 4D, under visible light irradiation of the as-prepared g-C_3_N_4_/0.50 wt% CuS photocatalyst, the photodegradation rate of MO was slightly suppressed by the addition of ·OH radical scavenger (*t*-BuOH, 1 mM), which reveals that ·OH radicals are not the main active species for the photodegradation of MO in current photocatalytic systems. However, in the presence of the ·O_2_^−^ radical scavenger (BQ, 1 mM) and the hole scavenger (Na_2_EDTA, 1 mM), the photodegradation rate of MO was decelerated significantly, with the photocatalytic degradation rate being reduced. It means that the ·O_2_^−^ and holes play the major roles in the photodegradation of MO over the as-prepared g-C_3_N_4_/0.50 wt% CuS photocatalyst under visible light irradiation.

**Fig. 6:**
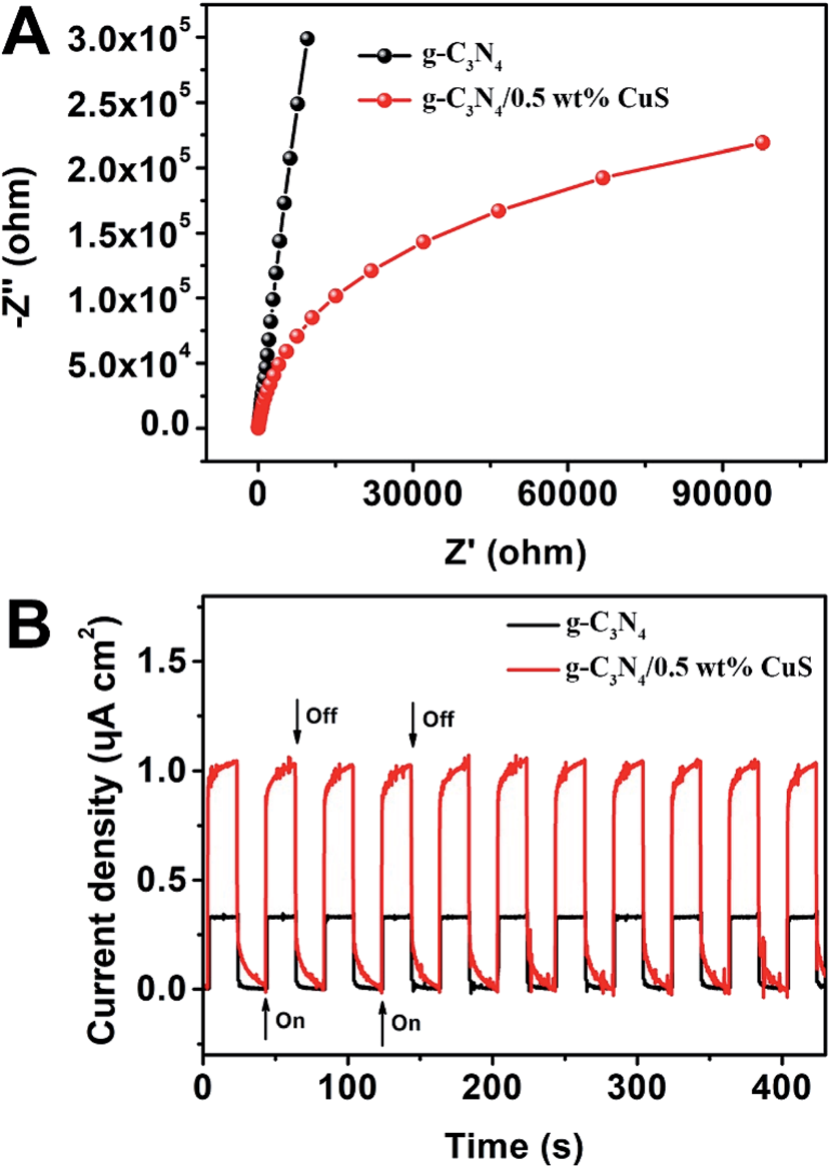
(A) EIS Nyquist plots of g-C_3_N_4_ and g-C_3_N_4_/0.5 wt% CuS. (B) Transient photocurrent density response of g-C_3_N_4_ and g-C_3_N_4_/0.5 wt% CuS during on/off cycles under 0.2 V bias *vs.* Ag/AgCl in a 0.2 M Na_2_SO_4_ electrolyte solution under solar simulator irradiation.

Photoelectrochemical (PEC) measurements, including electrochemical impedance spectroscopy (EIS) and transient photocurrent responses, of g-C_3_N_4_ and g-C_3_N_4_/0.5 wt% CuS samples are shown in Fig. 6. The high frequency region of Nyquist plots, providing useful information on charge transfer resistance, are shown in Fig. 6A. The arc radius on the EIS Nyquist plots of the g-C_3_N_4_/0.5 wt% CuS is smaller than that of g-C_3_N_4_, indicating that the loading of CuS NPs can reduce the charge transfer resistance and thus accelerate the interfacial charge transfer. Furthermore, the transient photocurrent measurement was carried out during light on–off cycles (Fig. 6B) to assess the charge carrier generation and transfer performance in the photoreaction system. The saturation photocurrent densities remained constant with light on, and immediately dropped to nearly zero once the light was switched off. The photocurrent density over g-C_3_N_4_/0.5 wt% CuS (∼1.05 μA cm^−2^) is about 3.2 times higher than that of the g-C_3_N_4_ (∼0.33 μA cm^−2^). The increased photocurrent confirms that the loaded CuS NPs could facilitate the separation and prolong the lifetime of the photoinduced charge carriers, which is responsible for the enhanced photocatalytic activities in water splitting and MO degradation. Moreover, the almost unchanged photocurrent response during repeated light on–off cycles is another evidence of the excellent stability of the as-prepared g-C_3_N_4_/CuS photocatalysts.

**Fig. 7:**
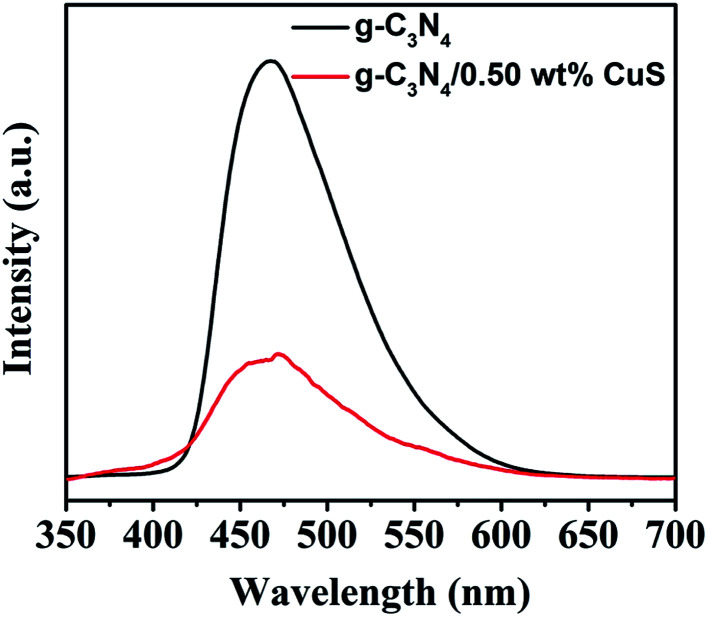
Room temperature PL spectra of g-C_3_N_4_, g-C_3_N_4_/0.5 wt% CuS under the excitation wavelength of 350 nm.

The PL spectrum is widely employed to evaluate the separation efficiency of charge carriers in photocatalysts. The PL spectra of g-C_3_N_4_ and g-C_3_N_4_/CuS samples are shown in Fig. 7. After 300 nm excitation, g-C_3_N_4_ and g-C_3_N_4_/CuS samples exhibit broad emission peaks in the range of 420–600 nm, representing that the photo-excited electrons recombine with holes. However, the PL intensity decreases after the introduction of CuS NPs, which indicates that the charge carries recombination in g-C_3_N_4_/0.5 wt% CuS was largely suppressed compared with g-C_3_N_4_.

### 3.3 Possible photocatalytic mechanism

**Fig. 8:**
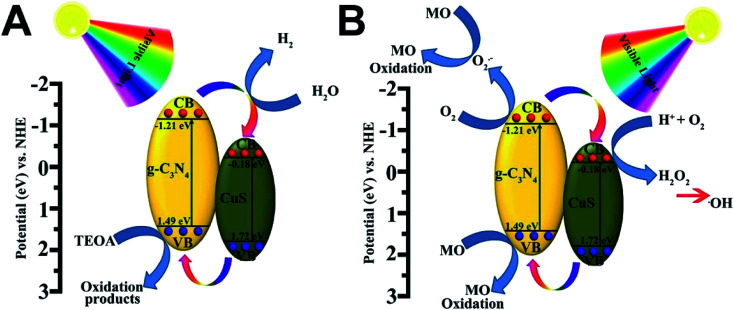
Schematic diagram illustrating (A) the photocatalytic degradation of MO and (B) the photocatalytic H_2_ production mechanisms over g-C_3_N_4_/CuS material under visible light irradiation.

Based on the above results, the superior photocatalytic performance of the g-C_3_N_4_/CuS composites can be assigned to the interfacial transfer of photogenerated electrons and holes between g-C_3_N_4_ and CuS, which leads to effective charge separation on both parts. We illustrate a possible visible light photocatalytic mechanism of H_2_ production and MO degradation in Fig. 8. For pure g-C_3_N_4_ and CuS, the photogenerated electron–hole pairs in both photocatalysts tend to recombine and only a small fraction of charge carriers could participate in the photocatalytic reaction because both pure photocatalysts showed less photocatalytic activities compared with composite materials. After heterojunction formation, g-C_3_N_4_ and CuS were excited simultaneously when the g-C_3_N_4_/CuS composites were irradiated with visible light, leading to generation of electron–hole pairs in the conduction band (CB) and valence band (VB), respectively. Since the CB of g-C_3_N_4_ (−1.21 eV *vs.* NHE) is more negative than that of CuS (−0.18 eV *vs.* NHE),^22,25^ the photoinduced electrons on the CB of g-C_3_N_4_ would easily transfer to the CB of CuS, and the photoinduced hole on the VB of CuS would easily transfer to the VB of g-C_3_N_4_, which both would restrain the photoinduced electron–hole pairs recombination and also prolong the reaction time, thereby improving the efficiency of H_2_ production from water and photodecomposition of MO. As a result, these electrons have longer time to reduce H^+^ to H_2_ on the CuS surface. In the meantime, the holes on the VB of the g-C_3_N_4_ reacted with the sacrificial agent (triethanolamine) (Fig. 8A). In the case of photocatalytic degradation of MO (Fig. 8B), the electrons on the CB of g-C_3_N_4_ could react with O_2_ to form ·O_2_^−^ radicals and the holes on the VB of g-C_3_N_4_ are considered as the active species. While the electrons on the CB of CuS is high enough to react with O_2_ to generate H_2_O_2_ for the reduction potential of O_2_/H_2_O_2_ is 0.695 eV/NHE.^22^ This possible photocatalytic degradation mechanism of g-C_3_N_4_/CuS composite was also proved by using the active species trapping experiment before.

## 4. Conclusion

In summary, highly active g-C_3_N_4_/CuS composites have been successfully prepared *via* the *in situ* hydrothermal method. The resulting composites photocatalysts exhibits significantly enhanced photocatalytic activities for H_2_ production and MO degradation under visible-light irradiation in comparison with the pure g-C_3_N_4_ and CuS. The g-C_3_N_4_/0.50 wt% CuS composite shows the best photocatalytic H_2_ production rate of 11.80 μmol h^−1^, which is about 2.7 times higher than that of pure g-C_3_N_4_. In addition, the g-C_3_N_4_/0.50 wt% CuS composite presents the superior degradation efficiency of MO and reuse ability. This work not only introduces a simple strategy to *in situ* grow metal sulfide NPs on carbon nitrides surfaces, but also provides useful insights into the development of low-cost CuS as a substitute for noble materials in the photocatalysis *via* a facile method based on g-C_3_N_4_.

## Conflicts of interest

There are no conflicts to declare.

## Supplementary Material

RA-009-C9RA03532J-s001
