# Supplementary material for: In situ growth of CuS nanoparticles on g-C3N4 nanosheets for H2 production and the degradation of organic pollutant under visible-light irradiation
Source: RSC Adv. 2019 Aug 15;9(44):25638–46. doi: 10.1039/c9ra03532j (PMC9070010; doi:10.1039/c9ra03532j)
Supplement: RA-009-C9RA03532J-s001 [file RA-009-C9RA03532J-s001.pdf]

Electronic Supplementary Material (ESI) for RSC Advances.
This journal is © The Royal Society of Chemistry 2019

Electronic supplementary information (ESI)

In-situ growth of CuS nanoparticles on g-C_3N_4 nanosheets for H_2 production and the degradation of organic pollutant under visible-light irradiation

Zhenhe Xu*[a], Baotong Xu[a], Kun Qian[a], Zheng Li[a], Fu Ding*[a], Miaomiao Fan[b], Yaguang Sun[a], Yu Gao*[a]

[a]*The Key Laboratory of Inorganic Molecule-Based Chemistry of Liaoning Province, College of Applied Chemistry, Shenyang University of Chemical Technology, Shenyang,110142, China*

[b]*Liaoyang Institure for Drug Control, Liaoyang, 111000, China*

E-mail: xuzh@syuct.edu.cn (Z.H. Xu); dingfu@syuct.edu.cn (F. Ding); gaoy777@126.com (Y. Gao)

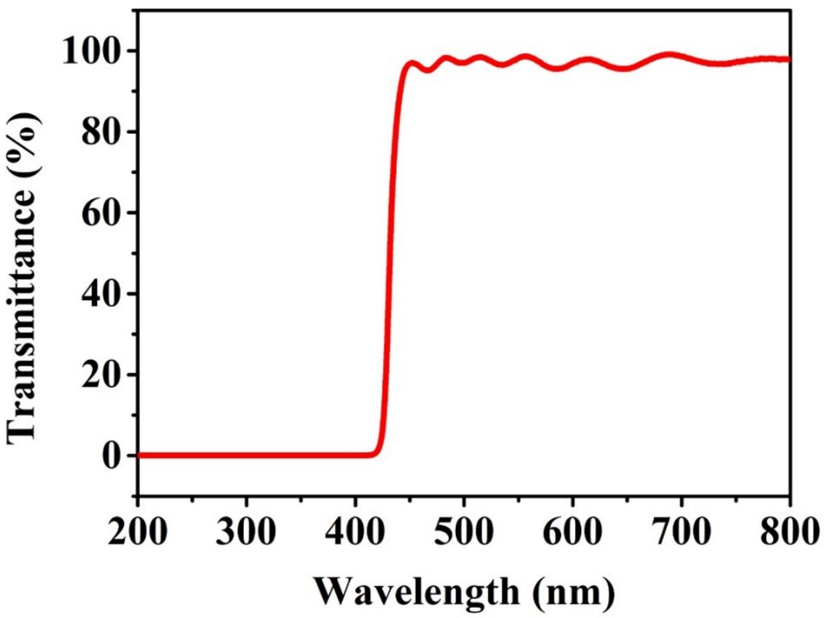

Fig. S1 Transmission spectra of a 420 nm cut-off filter used for photocatalytic measurements.

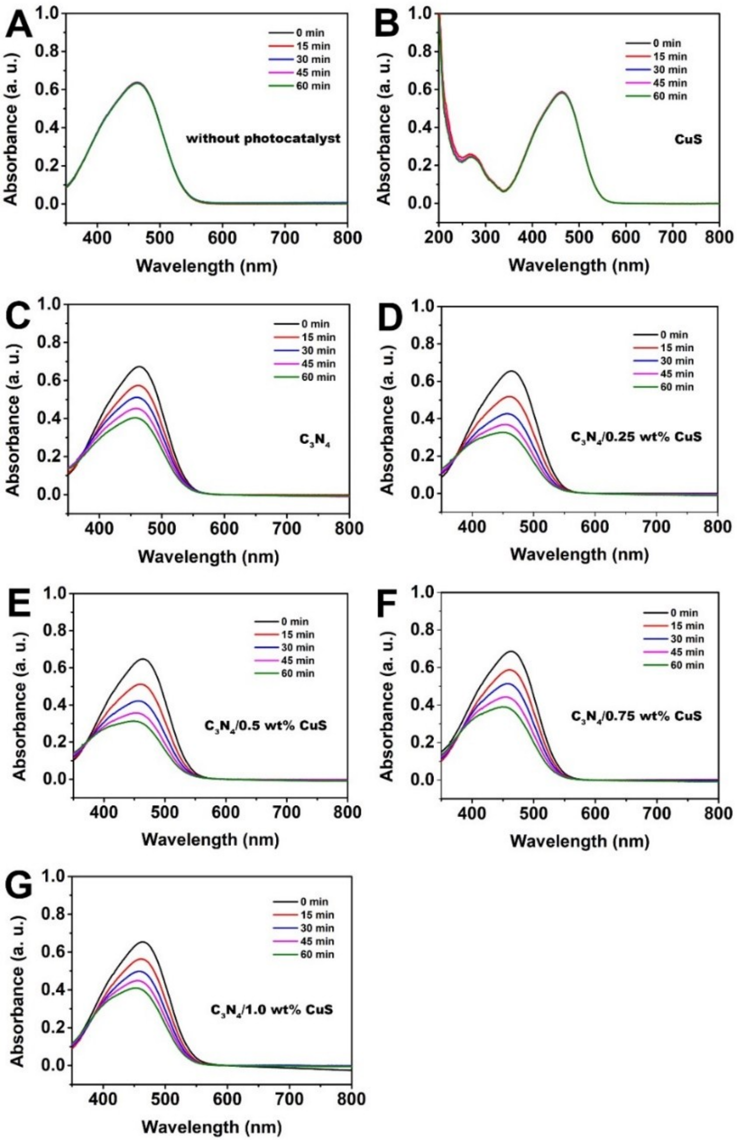

Fig. S2 UV-vis absorption spectra of MO solution separated from the suspensions containing (A) without photocatalyst, (B) CuS, (C) C_3N_4, (D) C_3N_4/0.25 wt% CuS, (E) C_3N_4/0.5 wt% CuS, (F) C_3N_4/0.75 wt% CuS, and (G) C_3N_4/1.0 wt% CuS.
